# Increased Soluble Epoxide Hydrolase in Human Gestational Tissues from Pregnancies Complicated by Acute Chorioamnionitis

**DOI:** 10.1155/2019/8687120

**Published:** 2019-12-05

**Authors:** Tai-Ho Hung, Szu-Fu Chen, Chun-Hu Wu, Chuan-Chi Kao, Chung-Pu Wu

**Affiliations:** ^1^Department of Obstetrics and Gynecology, Taipei Chang Gung Memorial Hospital, Taiwan; ^2^Department of Obstetrics and Gynecology, Keelung Chang Gung Memorial Hospital, Taiwan; ^3^College of Medicine, Chang Gung University, Taoyuan, Taiwan; ^4^Department of Physical Medicine and Rehabilitation, Cheng Hsin General Hospital, Taipei, Taiwan; ^5^Graduate Institute of Life Sciences, National Defense Medical Center, Taipei, Taiwan; ^6^Graduate Institute of Biomedical Sciences, Department of Physiology and Pharmacology and Molecular Medicine Research Center, College of Medicine, Chang Gung University, Taoyuan, Taiwan

## Abstract

Chorioamnionitis (CAM) is primarily a polymicrobial bacterial infection involving chorionic and amniotic membranes that is associated with increased risk of preterm delivery. Epoxyeicosatrienoic acids (EETs) are eicosanoids generated from arachidonic acid by cytochrome P450 enzymes and further metabolized mainly by soluble epoxide hydrolase (sEH) to produce dihydroxyeicosatrienoic acids (DHETs). As a consequence of this metabolism of EETs, sEH reportedly exacerbates several disease states; however, its role in CAM remains unclear. The objectives of this study were to (1) determine the localization of sEH and compare the changes it undergoes in the gestational tissues (placentas and fetal membranes) of women with normal-term pregnancies and those with pregnancies complicated by acute CAM; (2) study the effects of lipopolysaccharide (LPS) on the expression of sEH in the human gestational tissues; and (3) investigate the effect of 12-(3-adamantan-1-yl-ureido)-dodecanoic acid (AUDA), a specific sEH inhibitor, on LPS-induced changes in 14,15-DHET and cytokines such as interleukin- (IL-) 1*β* and IL-6 in human gestational tissues *in vitro* and in pregnant mice. We found that women with pregnancies complicated by acute CAM had higher levels of sEH mRNA and protein in fetal membranes and villous tissues compared to those in women with normal-term pregnancies without CAM. Furthermore, fetal membrane and villous explants treated with LPS had higher tissue levels of sEH mRNA and protein and 14,15-DHET than those present in the vehicle controls, while the administration of AUDA in the media attenuated the LPS-induced production of 14,15-DHET in tissue homogenates and IL-1*β* and IL-6 in the media of explant cultures. Administration of AUDA also reduced the LPS-induced changes of 14,15-DHET, IL-1*β*, and IL-6 in the placentas of pregnant mice. Together, these results suggest that sEH participates in the inflammatory changes in human gestational tissues in pregnancies complicated by acute CAM.

## 1. Introduction

Chorioamnionitis (CAM) is primarily a polymicrobial bacterial infection involving chorionic and amniotic membranes and amniotic fluid and is implicated in 0.5% to 10% of all pregnancies [1]. A number of studies have demonstrated that CAM increases the risk of preterm labor and subsequent preterm delivery [2, 3]. In addition, CAM is associated with adverse neonatal outcomes including cerebral palsy and bronchopulmonary dysplasia [4, 5].

Eicosanoids are a group of lipid mediators generated from arachidonic acid (AA) by the activity of cyclooxygenases (COX), lipoxygenases (LOX), and cytochrome P450 (CYP450) enzymes. Previous studies—mostly focusing on the COX and LOX pathways—have identified the essential roles of AA metabolism and eicosanoids in the pathophysiology of CAM. These include increased levels of AA in the placentas of women with CAM and preterm delivery compared to women with preterm delivery but without CAM [6]; upregulation of COX-2 and prostaglandin (PG) G/H synthase, along with certain inflammatory genes, in the amnion and choriodecidua of women with CAM [7]; increased production of PGE_2_, PGF_2*α*_, and leukotriene B_4_ (LTB_4_) from the placentas and fetal membranes in women with CAM than in those without CAM [8, 9]; and greater concentrations of PGs and LTB_4_ in the amniotic fluid of women with intra-amniotic infections than in women without intra-amniotic infections [10–12]. Nevertheless, the role of AA metabolism via the CYP450 pathway in human gestational tissues of pregnancies complicated by acute CAM remains unclear.

Unlike most metabolites of the COX and LOX pathways, metabolites of the CYP450 enzymes, mainly epoxyeicosatrienoic acids (EETs) and dihydroxyacids, have primarily vasodilatory, antihypertensive, anti-inflammatory, and natriuretic properties [13, 14]. EETs are further metabolized by soluble epoxide hydrolase (sEH), which adds water across the epoxide to give the corresponding dihydroxyeicosatrienoic acids (DHETs) [14, 15]. As a consequence of its metabolism of EETs, sEH reportedly contributes significantly to the pathogenesis of several disease states such as diabetes, hypertension, and pain [16]. Indeed, increasing evidence indicates that the inhibition of sEH increases levels of EETs, which have anti-inflammatory effects and can prevent the development of hypertension, atherosclerosis, heart failure, fatty liver, and multiple organ fibrosis [14, 16, 17].

EETs, DHETs, and sEH have been detected in many human tissues including the placentas and fetal membranes [18, 19]. Considering its central role in the metabolism of EETs, we surmised that there is dysregulation of sEH in human gestational tissues (placental villi and fetal membranes) in pregnancies complicated by acute CAM. Therefore, the objectives of this study were to (1) determine and compare the localization and changes of sEH in human gestational tissues between women with normal-term pregnancies and those with pregnancies complicated by acute CAM; (2) study the effects of lipopolysaccharide (LPS)—an endotoxin found in gram-negative bacteria that is reportedly elevated in the amniotic fluid and choriodecidua during acute CAM—on the expression of sEH in human gestational tissues *in vitro*; and (3) investigate the effect of 12-(3-adamantan-1-yl-ureido)-dodecanoic acid (AUDA)—a specific sEH inhibitor—on LPS-induced changes in 14,15-DHET and cytokines such as interleukin-1*β* and IL-6 in human gestational tissues *in vitro* and in pregnant mice.

## 2. Materials and Methods

Conduction of this study was approved by the Institutional Review Board of Chang Gung Memorial Hospital (No. 201601866B0 and No. 201802304B0). All placental samples were collected after the subjects enrolled herein provided written informed consent for the use of the samples. Unless otherwise indicated, the reagents used in the study were purchased from Sigma-Aldrich (St. Louis, MO, USA).

### 2.1. Placental Collection and Sampling

Human placentas and attached fetal membranes were collected from 20 normal-term pregnant women experiencing cesarean delivery due to previous section or fetal malpresentation and 16 women with cesarean delivery for acute CAM. We used the following criteria to identify CAM in the subjects of this study: maternal fever (body temperature higher than 38°C), the rupture of membranes, and the presence of one of the following conditions: leukocytosis (white blood cell counts higher than 12,000/*μ*L), elevated serum levels of C-reactive protein (>5 mg/dL), and fetal tachycardia (a baseline fetal heart rate > 160 beats/min) followed by histological confirmation (defined as extensive infiltration of neutrophils from the subchorionic space throughout the chorion) [20].

After the placenta was delivered, fetal membranes (amnion and choriodecidua) were separated from the placenta by blunt dissection under sterile conditions. Furthermore, villous tissues were randomly sampled from five distinct sites from the maternal side using a transparent sheet bearing a systematic array of sampling windows; each site was middistance between the cord insertion and the periphery of the placenta and midway between the chorionic and basal plates. After a brief rinse in cold phosphate-buffered saline, several tissue fragments of fetal membrane rolls and villous samples were either fixed in 4% paraformaldehyde or snap frozen in liquid nitrogen. The remaining fragments were placed in culture medium (medium-199 with 25 mM HEPES, Earle's salts, and L-glutamine supplemented with 5% heat-inactivated fetal bovine serum, 100 U/mL penicillin, 100 *μ*g/mL streptomycin, and 0.25 *μ*g/mL amphotericin B) and transferred to the laboratory on ice for individual experiments. All placental samples were collected and processed within 10 minutes of the delivery.

### 2.2. Fetal Membrane and Villous Explant Culture

Fetal membrane and villous explant cultures were performed as described previously [21, 22]. Briefly, under sterile laboratory conditions, membranes were checked for integrity to ensure that the amnion and chorion remained attached to one another. The membranes were rinsed with culture medium to remove residual adherent blood, cut into approximately 6 × 6 cm^2^ pieces, bound together with Orthodontic Elastic Rubber Bands (5/16 inch; ORMCO Z-pak elastics, Ormco Corporation, Glendora, CA, USA), and placed on the upper chamber of a Corning Transwell® device (24 mm diameter without synthetic membrane; Corning, NY, USA). The inserts were placed in 6-well culture plates to create two distinct chambers containing 2 mL of culture medium on both sides of the mounted membranes. The amniotic side of the membranes faced the inner chamber of the Transwell® device; excess tissue extending beyond the elastic band was trimmed with a scalpel.

For the preparation of villous explants, villous samples were dissected into pieces weighing 5–10 mg and placed in culture medium; six to eight such pieces were suspended at the gas-liquid interface in individual Costar Netwell® inserts (24 mm diameter, 500 *μ*m mesh) suspended in 3 mL of culture medium. Both fetal membranes and villous explants were equilibrated in culture media at 37°C and 5% CO_2_ in a humidified incubator overnight. The medium was refreshed the following day, and the explants were cultured under individual experimental conditions.

### 2.3. Explant Culture with or without LPS and AUDA Administration

Based on our preliminary concentration-response experiments, LPS from *Escherichia coli 026:B6* (Sigma-Aldrich) at a concentration of 10 *μ*g/mL and AUDA (Cayman Chemical, Ann Arbor, MI, USA) at a concentration of 10 *μ*M were used to assess their effects on the changes in sEH and 14,15-DHET in tissue samples and the production of cytokines including interleukin- (IL-) 1*β* and IL-6 in the medium for explant culture experiments (Supplementary Figures [Supplementary-material supplementary-material-1] and [Supplementary-material supplementary-material-1]). After overnight rest, fetal membrane and villous explants were incubated in fresh media with or without LPS in the absence or presence of AUDA for 24 hours. Thereafter, the medium was recovered from both sides of the membranes from the two-chamber culture system or from the villous explant cultures and stored at -80°C. After collection of medium, amnion, choriodecidua, and villous samples were removed from the Transwell® frame and snap frozen in liquid nitrogen for subsequent analysis. Ten placentas were used to study the effect of LPS on the transcription and translation of sEH in fetal membranes and villous explants, and eight were used to study the effect of AUDA on LPS-induced changes in the expression of 14,15-DHET in the tissue homogenates and IL-1*β* and IL-6 in the media of explant cultures, respectively.

### 2.4. Animal Experiments

Time-pregnant C57BL/6 mice ordered from BioLASCO (Taipei, Taiwan) were used in this study. Mice were allowed free access to water and maintained on a 12 h/12 h dark cycle at a controlled temperature (22-25°C) and humidity (40-60%). All the experimental protocols were approved by the Institutional Animal Care and Use Committee at Cheng Hsin General Hospital (animal permit number CHGH-105-10), and all animals were cared for in accordance with the Guide for the Care and Use of Laboratory Animals published by the US National Institutes of Health (NIH Publication No. 85-23, revised 1996).

On day 15 gestation, pregnant mice received a single intraperitoneal injection of LPS (300 *μ*g/kg in 0.1 mL saline) derived from *Escherichia coli O111:B4*. An equivalent saline injection was administered for the control group (sham control). Ten minutes after LPS administration, mice were randomized to receive an intraperitoneal injection of either AUDA at a concentration of 20 mg/kg or an equal volume of vehicle (5% DMSO). Six hours after LPS administration, pregnant mice were terminally anaesthetized using 5% isoflurane (2 L/min O_2_ flow rate), and the uterus was exteriorized via a midline laparotomy to identify individual gestational sacs. The gestational sacs were then opened, and individual placentas were collected, snapped frozen in liquid nitrogen, and stored at -80°C for further assessment. Eight mice for each group were used for this experiment.

### 2.5. Tissue Integrity Assessment

Fresh villous tissues and membranes from five different placentas were incubated in phenol red-free culture medium to monitor tissue integrity after LPS stimulation with or without AUDA, as assessed by the release of lactate dehydrogenase into the culture medium, using a commercial kit (Cytotoxicity Detection Kit, Roche Diagnostics GmbH, Mannheim, Germany) according to the manufacturer's instructions.

### 2.6. Immunohistochemistry

Immunohistochemistry for sEH, cytokeratin 7 (CK7), and CD68 was performed as previously described [20]. Briefly, after quenching endogenous peroxidase activity and blocking nonspecific binding, sections were reacted with mouse anti-human sEH (A-5) monoclonal antibody (1 : 10 dilution; catalog no. SC-166961; Santa Cruz Biotechnology, Inc. Dallas, TX, USA), anti-human CK7 monoclonal antibody (1 : 1000 dilution, clone OV-TL 12/30, Dako Omnis, Agilent, Santa Clara, CA, USA), or anti-human CD68 monoclonal antibody (1 : 1000 dilution, clone KP1, Dako Omnis) at 4°C overnight. Further processing for colorimetric detection was according to the instructions for the VECTASTAIN Elite ABC Kit (Vector Laboratories, Burlingame, CA, USA) using diaminobenzidine as the peroxidase substrate. The specificity of a staining reaction was assessed in several control procedures, including omission of the primary antibody or substitution of the primary antibody with nonimmune mouse isotypic IgG. Tissue sections from C57BL/6 mice livers were used as the positive controls for sEH immunostaining [23]. Sections were viewed and photographed under a differential interference contrast microscope (Nikon Eclipse 80i, Nikon Corporation, Tokyo, Japan).

### 2.7. Western Blot

Western blotting was performed as previously detailed [24]. Fifty to 100 micrograms of cytosolic proteins was separated by 8-12% SDS-PAGE, transferred to nitrocellulose membranes, and probed with the mouse anti-human sEH (A-5) monoclonal antibody (1 : 200 dilution; catalog no. SC-166961; Santa Cruz Biotechnology, Inc.) at 4°C overnight. The relative intensities of the protein signals were normalized to the intensities of the *β*-actin (clone AC-15, Sigma-Aldrich) signals, and the band densities were quantified by densitometric analysis using ImageJ software (National Institutes of Health, Bethesda, MD, USA; https://rsb.info.nih.gov/ij/). Tissue homogenates from C57BL/6 mice livers were used as the positive controls [23]. The sample in the vehicle control group with the lowest sEH/*β*-actin ratio was used as the reference for comparison, and the data are presented as fold changes.

### 2.8. Real-Time Quantitative PCR

Real-time quantitative PCR analysis was performed as previously described [21]. Real-time quantitative PCR analysis was performed with an ABI PRISM 7900 sequence detector (Applied Biosystems). Assay-on-Demand TaqMan primers and probes for human *EPHX2* (Hs00157403_m1) from Applied Biosystems were used. 18S ribosomal RNA (Hs99999901_s1) was used as an endogenous control. Thermal cycling is initiated with a 2-minute incubation at 50°C, followed by a first denaturation step of 10 minutes at 95°C, and then 40 cycles of 95°C for 15 seconds and 60°C for 1 minute. All samples were analyzed on the same run, and each sample was run in triplicate. Relative quantities of *EPHX2* mRNA and 18S ribosomal RNA were calculated by the comparative threshold cycle (Ct) method. Briefly, the Ct value of sEH gene was subtracted from that of 18S ribosomal RNA (expressed as ΔCt), which acted as a standard for the amount of RNA template and efficiency of reverse transcription. Then, the ΔCt values of samples treated with LPS, AUDA, or both were normalized to the sample with the largest ΔCt value in the vehicle control group. The resulting change in ΔCt values (expressed as ΔΔCt) was converted to a linear form using 2^(−ΔΔCt)^ and used in subsequent statistical analysis.

### 2.9. Enzyme-Linked Immunosorbent Assay (ELISA)

After individual experiments, villous tissues and fetal membrane fragments from explant cultures and placentas from mice were homogenized and centrifuged and protein concentrations in the supernatants were determined. The levels of 14,15-DHET in the tissue homogenates, the levels of IL-1*β* and IL-6 in the media of explant culture experiments, and tissue levels of 14,15-DHET, IL-1*β*, and IL-6 in the placentas of mice were determined using commercial ELISA kits according to the manufacturers' instructions. The following ELISA kits were used in this study: 14,15-DHET ELISA Kit (catalog no. DH2; Detroit R&D, Inc., Detroit, MI, USA), human IL-1*β* Quantikine HS ELISA Kit (catalog no. HSLB00D; R&D Systems, Minneapolis, MN, USA), human IL-6 Quantikine ELISA Kit (catalog no. HS6050; R&D Systems), mouse IL-1*β* Quantikine HS ELISA Kit (catalog no. MLB00C; R&D Systems), and mouse IL-6 Quantikine ELISA Kit (catalog no. M6000B; R&D Systems). For IL-1*β* and IL-6 measurements, all samples and standards were assayed in duplicate and the results were normalized per mg protein. For 14,15-DHET measurements, all samples and standards were assayed in duplicate; the sample in the vehicle control group with the lowest level was used as the reference for comparison and the data are presented as fold changes.

### 2.10. Statistical Analysis

For maternal and pregnancy characteristics, numerical data are presented as the mean ± SD or median and interquartile range when data were not normally distributed; categorical variables are presented as a number and rate (%). For western blot analysis, real-time qPCR, and ELISA, data are presented as the mean ± SEM. Data were analyzed and plotted using Prism 7 for Mac OS X, version 5.0d (GraphPad Software, Inc., La Jolla, CA, USA). Differences between two groups were computed with the Mann-Whitney *U*-test or Student's *t*-test. Differences among groups of three or more were determined by one-way analysis of variance (ANOVA) followed by Bonferroni's post hoc test. A *P* value of <0.05 was considered statistically significant.

## 3. Results

### 3.1. Characteristics of the Study Population

The characteristics of the study population are shown in Table 1. Compared to normal pregnant women, women with pregnancies complicated by acute CAM had higher body temperature, white blood cell counts, proportions of segmented neutrophils, and baseline fetal heart rate. Furthermore, microbial growth in the amniotic fluid during cesarean delivery was found in 7 of the 16 (44%) patients with acute CAM.

### 3.2. Localization of sEH in the Fetal Membranes from Normal Pregnant Women and Women with Acute CAM

**Figure 1:**
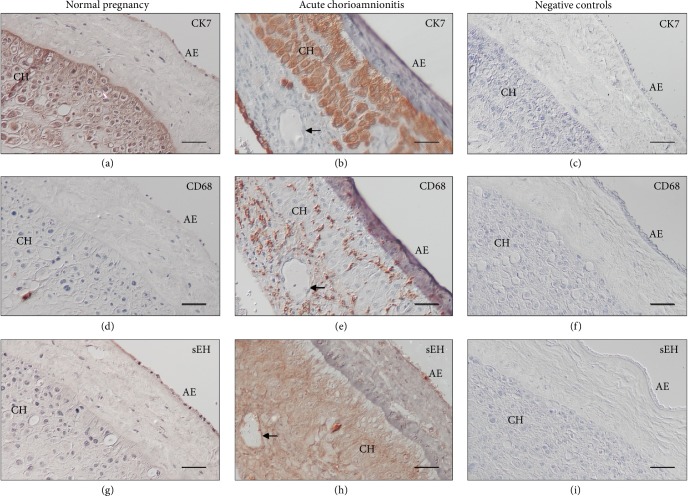
Immunoreactivity for sEH in fetal membranes from normal pregnant women and women with pregnancies complicated by acute CAM. Immunostaining of CK7 delineates the localization of amniotic epithelium and trophoblast cells in choriodecidua (a, b). Compared to normal pregnancy, fetal membranes from women with acute CAM had increased infiltration of monocytes/macrophages, as demonstrated by positive anti-CD68 immunostaining, within the amnion and choriodecidua (d, e). In normal pregnancy, immunostaining of sEH was observed in the amniotic epithelium (g). The immunostaining of sEH was generally more intense, both in the amnion and choriodecidua, in women with acute CAM compared with that in normal pregnant women (h). There was no staining observed in the negative controls when the primary antibody was substituted with nonimmune IgG (c, f, and i). Scale bar = 50 *μ*m. AE, amniotic epithelium; CH, choriodecidua; arrows, endothelium.

In normal-term pregnancy, immunostaining of sEH was observed in the amniotic epithelium (Figure 1(g)). The immunostaining of sEH was generally more intense, both in the amnion and choriodecidua, in women with acute CAM than in normal pregnant women (Figure 1(h)). In parallel with the increased immunostaining of sEH in fetal membranes from pregnancies complicated by acute CAM, there was also increased infiltration of monocytes/macrophages, as demonstrated by positive anti-CD68 immunostaining, within the amnion and choriodecidua (Figures 1(d) and 1(e)).

### 3.3. Increased sEH Protein and mRNA Levels in Fetal Membranes from Women with Pregnancies Complicated by Acute CAM

**Figure 2:**
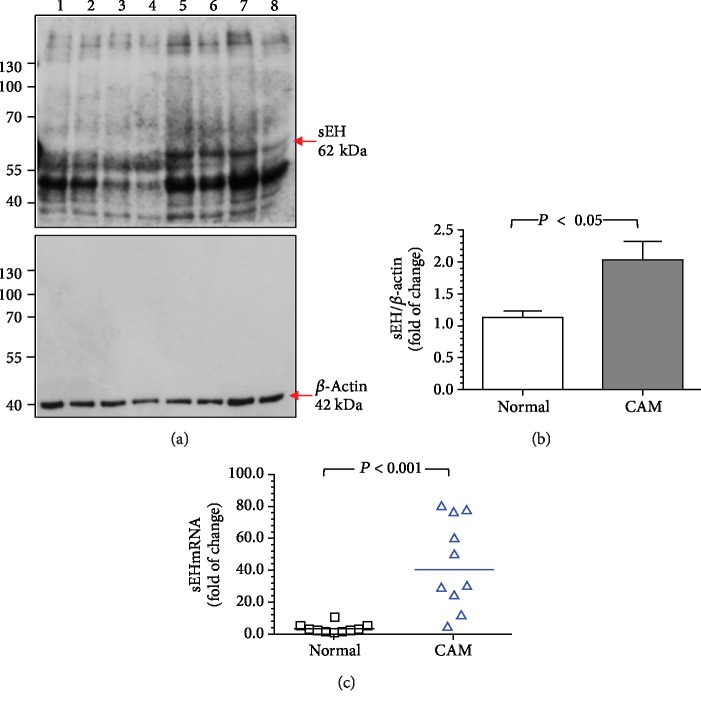
The levels of sEH protein and mRNA in fetal membranes from normal pregnant women and women with pregnancies complicated by acute CAM. Compared to normal pregnant women, women with acute CAM had significantly higher levels of sEH protein (a, b) and mRNA (c) in fetal membrane homogenates. (a) Representative blots showing the expression of sEH between normal pregnant women and women with acute CAM. Lanes 1-4, fetal membrane homogenates from 4 women with normal pregnancy; lanes 5-8, homogenates from 4 women with pregnancies complicated by acute CAM. *β*-Actin was used to normalize loading variability. Molecular weight markers are shown at the left side of the blots. (b) Data are presented as mean ± SEM and *P* value is based on Student's *t*-test. (c) Horizontal bars represent the median values, and *P* value is based on the Mann-Whitney *U*-test. A total of 10 women with normal pregnancy and 10 women with pregnancies complicated by acute CAM were examined.

Compared with normal pregnant women, women with pregnancies complicated by acute CAM had significantly higher levels of sEH protein and mRNA in fetal membranes (Figure 2).

### 3.4. Localization of sEH in the Villous Tissues from Normal Pregnant Women and Women with Acute CAM

**Figure 3:**
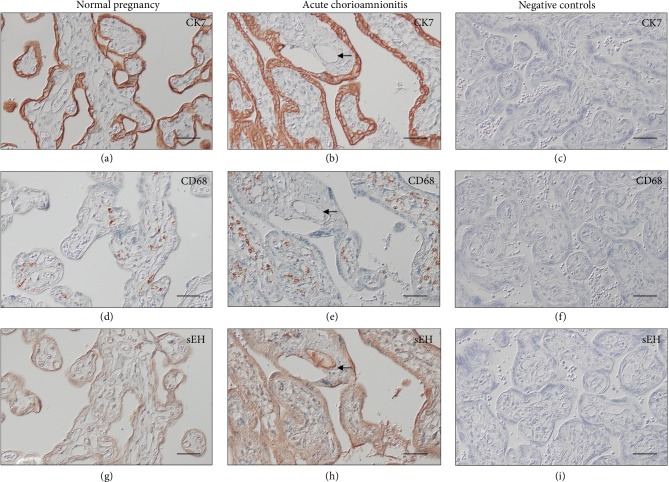
Immunoreactivity for sEH in villous tissues from normal pregnant women and women with pregnancies complicated by acute CAM. Immunostaining of CK7 delineates the localization of cytotrophoblasts and syncytiotrophoblasts (a, b). Compared to normal pregnancies, villous tissues from women with acute CAM had increased infiltration of monocytes/macrophages, as demonstrated by positive anti-CD68 immunostaining, within the stroma (d, e). In normal pregnancies, immunostaining of sEH was found mainly in the trophoblast layer and some stromal cells (g). The immunostaining of sEH was generally increased in women with acute CAM compared to normal pregnant women (h). In addition, strong sEH immunostaining was noted in the villous endothelium in women with acute CAM (arrows). There was essentially no staining at all on the negative controls when the primary antibody was substituted with nonimmune IgG (c, f, and i). Scale bar = 50 *μ*m.

As shown in Figure 3(g), immunoreactivity for sEH was found in the trophoblast layer of villous tissues obtained from normal pregnant women. A few stromal cells also reacted to the anti-sEH antibody. In villous samples from pregnancies complicated by acute CAM, the immunostaining of sEH was generally more intense—in particular in the trophoblast layer and villous endothelium—than in those from normal pregnancies lacking the symptoms and signs of CAM (Figure 3(h)). This change was associated with increased infiltration of monocytes/macrophages into the stroma of the villi (Figures 3(d) and 3(e)).

### 3.5. Increased sEH Protein and mRNA Levels in Villous Tissues from Women with Pregnancies Complicated by Acute CAM

**Figure 4:**
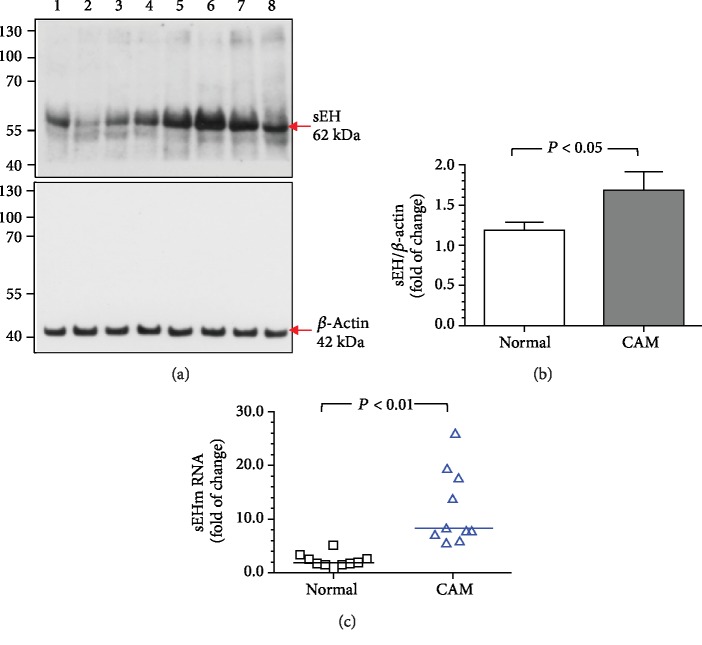
The levels of sEH protein and mRNA in villous tissues from normal pregnant women and women with pregnancies complicated by acute CAM. Compared with normal pregnant women, those with acute CAM showed significantly higher levels of sEH protein (a, b) and mRNA (c) in villous homogenates. (a) Representative blots showing the expression of sEH between normal pregnant women and those with acute CAM. Lanes 1-4, villous tissue homogenates from 4 women with normal pregnancy; lanes 5-8, homogenates from 4 women with pregnancies complicated by acute CAM. *β*-Actin was used to normalize loading variability. Molecular weight markers are shown at the left side of the blots. (b) Data are presented as mean ± SEM and *P* value is based on Student's *t*-test. (c) Horizontal bars represent the median values and *P* value is based on the Mann-Whitney *U*-test. A total of 10 women with normal pregnancy and 10 women with pregnancies complicated by acute CAM were examined.

Similarly, the villous samples of women with pregnancies complicated by acute CAM had significantly higher levels of sEH protein and mRNA than those of normal pregnant women (Figure 4).

### 3.6. LPS Led to Increased sEH mRNA and Protein Levels in Fetal Membrane and Villous Explants

**Figure 5:**
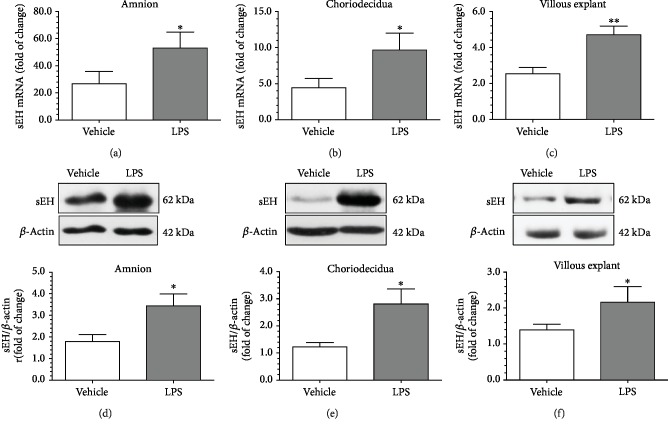
LPS led to increased sEH mRNA and protein levels in fetal membrane and villous explants. Compared to the vehicle controls, the administration of LPS to the culture medium led to a significant increase in the levels of sEH mRNA and protein in the amnion and choriodecidua, respectively, with the two-chamber culture system (a, b, d, and e). Similar effects of LPS on the expression of sEH was also observed in villous explants (c and f). *β*-Actin was used to normalize loading variability. Data are presented as mean ± SEM, *n* = 10 for each group. ^∗^*P* < 0.05 and ^∗∗^*P* < 0.01 compared with the vehicle control by Student's *t*-test.

The most well-recognized predisposing factor to acute CAM is ascending infection by microorganisms from the lower genital tract. We therefore studied the effect of bacterial endotoxin, LPS, on the expression of sEH in fetal membrane and villous explant cultures. Compared to the vehicle controls, administration of LPS in the culture medium significantly increased the levels of sEH mRNA and protein in the amnion and choriodecidua with the two-chamber culture system (Figures 5(a), 5(b), 5(d), and 5(e) and Supplementary Figures [Supplementary-material supplementary-material-1] and [Supplementary-material supplementary-material-1]). Similar effects of LPS on the expression of sEH were also observed in villous explant culture experiments (Figures 5(c) and 5(f) and Supplementary [Supplementary-material supplementary-material-1]).

### 3.7. Administration of AUDA Reduced the Tissue Levels of 14,15-DHET in Fetal Membrane and Villous Explants Treated with LPS

**Figure 6:**
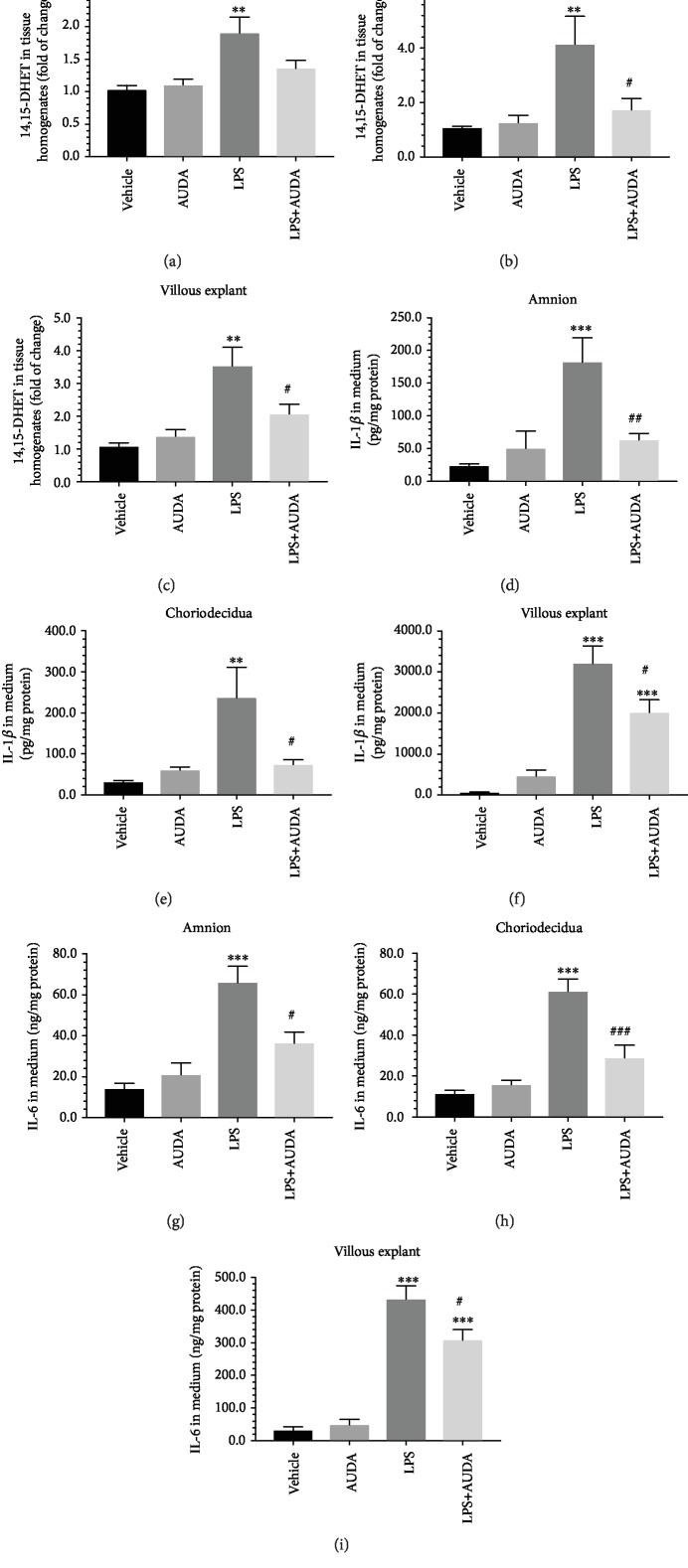
Administration of AUDA reduced the levels of 14,15-DHET in tissue homogenates and the levels of IL-1*β* and IL-6 in the culture media of fetal membrane and villous explants treated with LPS. LPS caused a significant increase in the tissue levels of 14,15-DHET in the amnion, choriodecidua, and villous explants compared to the vehicle controls (a–c). In contrast, the administration of AUDA reduced the changes in 14,15-DHET induced by LPS; however, only the differences in choriodecidua and villous explants reached statistical significance (b, c). Similarly, the levels of IL-1*β* and IL-6 in the media were significantly higher in fetal membrane and villous explants stimulated with LPS than in the vehicle controls (d-i). Administration of AUDA significantly attenuated the changes in IL-1*β* and IL-6 in the media caused by LPS treatment in fetal membrane and villous explants. Data are presented as mean ± SEM, *n* = 8 for each group. ^∗∗^*P* < 0.01 and ^∗∗∗^*P* < 0.001 compared with the vehicle controls; ^#^*P* < 0.05, ^##^*P* < 0.01, and ^###^*P* < 0.001 compared with explants treated with LPS, with one-way ANOVA followed by Bonferroni's post hoc test.

14,15-DHET is one of the major DHETs produced by sEH in human placentas; therefore, measurement of 14,15-DHET can be regarded as an indicator of the enzymatic activity of sEH. To study the changes of sEH activity in response to LPS stimulation with or without AUDA, we measured the tissue levels of 14,15-DHET in the homogenates of fetal membrane and villous explants, respectively. As shown in Figures 6(a)–6(c), LPS caused a significant increase in 14,15-DHET levels in the amnion, choriodecidua, and villous explants compared to the vehicle controls. In contrast, administration of AUDA reduced the changes in 14,15-DHET induced by LPS; the effects were more profound in choriodecidua and villous explants than in the amnion (Figures 6(b) and 6(c)).

### 3.8. Administration of AUDA Reduced the Levels of IL-1*β* and IL-6 in the Culture Media of Fetal Membrane and Villous Explants Treated with LPS

To further explore the role of sEH in the inflammatory response associated with LPS stimulation, we measured the levels of IL-1*β* and IL-6 in the media of fetal membrane and villous explants stimulated by LPS with or without AUDA. As expected, the levels of IL-1*β* and IL-6 in the media were significantly higher in fetal membrane and villous explants stimulated with LPS than in the vehicle controls (Figures 6(d)–6(i)). Again, administration of AUDA significantly attenuated the changes in IL-1*β* and IL-6 in the media caused by LPS treatment in fetal membrane (Figures 6(d), 6(e), 6(g), and 6(h)) and villous explants (Figures 6(f) and 6(i)).

### 3.9. Administration of AUDA Reduced the Levels of 14,15-DHET, IL-1*β*, and IL-6 in the Placentas of Mice Treated with LPS

**Figure 7:**
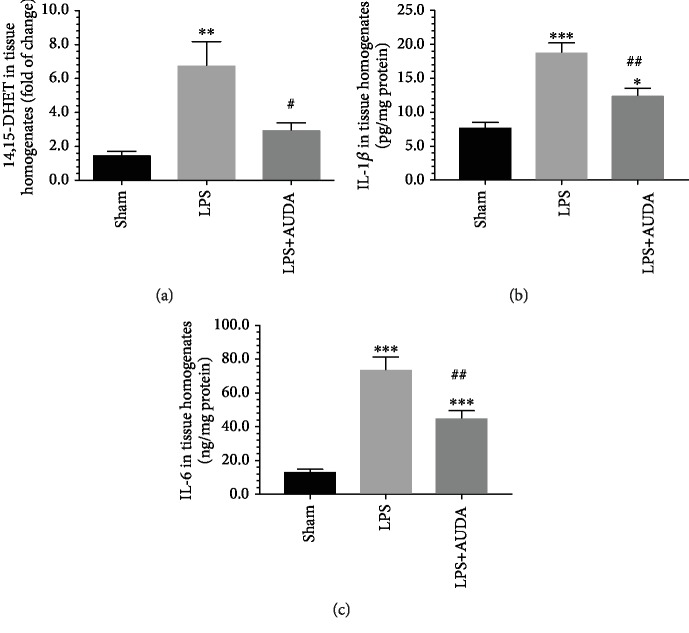
Administration of AUDA reduced the levels of 14,15-DHET, IL-1*β*, and IL-6 in the placentas of pregnant mice treated with LPS. Compared to the sham controls, pregnant mice treated with LPS had significantly higher levels of 14,15-DHET, IL-1*β*, and IL-6 in the placentas. Administration of AUDA significantly reduced the extent of these changes caused by LPS. Data are presented as mean ± SEM based on 8 mice for each group. ^∗∗^*P* < 0.01 and ^∗∗∗^*P* < 0.001 compared with the sham controls; ^#^*P* < 0.05 and ^##^*P* < 0.01 compared with mice treated with LPS, with one-way ANOVA followed by Bonferroni's post hoc test.

To verify the effects of AUDA on the changes of 14,15-DHET, IL-1*β*, and IL-6 associated with LPS stimulation, we compared the placental levels of 14,15-DHET, IL-1*β*, and IL-6 between pregnant mice treated with intraperitoneal injection of LPS in the absence or presence of AUDA. Compared to the sham controls, mice treated with LPS had significantly higher 14,15-DHET, IL-1*β*, and IL-6 levels in the placentas (Figure 7). Administration of AUDA reduced the extent of these changes caused by LPS, though the levels of 14,15-DHET, IL-1*β*, and IL-6 were still higher than that in the sham controls.

## 4. Discussion

Our results demonstrate that (1) women with pregnancies complicated by acute CAM had more intense immunostaining of sEH and higher levels of sEH mRNA and protein in fetal membranes and villous tissues than women with normal-term pregnancies without CAM; (2) administration of LPS to the media led to increased tissue levels of sEH mRNA and protein and 14,15-DHET both in fetal membrane and villous explants; (3) administration of AUDA to the media reduced the LPS-induced production of 14,15-DHET in tissue homogenates and IL-1*β* and IL-6 in the media of fetal membrane and villous explants; and (4) AUDA attenuated the levels of 14,15-DHET, IL-1*β*, and IL-6 in the placentas of pregnant mice treated with LPS. Together, these results suggest that sEH participates in the inflammatory changes in human gestational tissues in pregnancies complicated by acute CAM.

sEH expression has been detected in the gravid uterus and placenta including fetal membranes [18, 19, 25]; however, its role in these tissues remains elusive. Using myometrial strips obtained from normal pregnant women experiencing cesarean deliveries, Corriveau et al. found that administration of EETs or AUDA to the tissue bath significantly inhibited spontaneous myometrial contractile activities *in vitro* [25]. They further showed that the levels of sEH were greater in the myometrium of LPS-treated pregnant rats than in pregnant rats without LPS stimulation and that myometrial contractile activity induced by LPS was reduced upon administration of EETs or AUDA [18]. In contrast, women with preeclampsia have higher plasma and placental levels of EETs than normal pregnant women [19, 26, 27]. Furthermore, the expression of sEH was lower in preeclamptic placentas compared to normal placentas [19]. The current study expands upon previous findings of the presence of sEH in human gestational tissues and provides new evidence of the role of placental sEH in pregnancy complications including acute CAM.

The mechanisms underlying the regulation of sEH expression in human gestational tissues in pregnancies complicated by CAM and in response to LPS stimulation remain unclear. Our recent study shows that NF-*κ*B signaling is a possible pathway [23]. The sEH gene promoter region contains recognition sites for a number of transcription factors including NF-*κ*B [28]. NF-*κ*B is a key regulator of proinflammatory cytokine production in response to infection [29]. Indeed, increased NF-*κ*B activity has been detected in the placentas of pregnancies complicated by acute CAM compared to those without acute CAM [30]. Furthermore, activation of NF-*κ*B has been observed in placentas perfused with LPS *ex vivo* [31] and in villous and fetal membrane explants treated with LPS *in vitro* [32].

In addition to its effect on the induction of sEH, we found that LPS treatment simultaneously caused increased levels of 14,15-DHET in the tissue homogenates of fetal membrane and villous explants, suggesting an increased activity of sEH in these tissues. We also observed that the administration of AUDA reduced the levels of 14,15-DHET induced by LPS, confirming the inhibitory effect of AUDA on sEH activity. We further demonstrated that the administration of AUDA reduced the LPS-stimulated production of IL-1*β* and IL-6 in fetal membrane and villous explants and in pregnant mice. These findings suggest an association between the pharmacological inhibition of sEH and the reduced production of cytokines in response to LPS stimulation, though the mechanisms regulating these changes are not clear. Previous studies on other organ systems have shown that the administration of EETs or AUDA attenuated the phosphorylation of mitogen-activated protein (MAP) kinases such as c-Jun N-terminal kinases (JNK) and p38 [23, 33, 34]. MAP kinases have been reported to participate in the inflammatory response to LPS stimulation in the cells or tissues obtained from pregnant women including amnion cells [35], decidual cells [36], cytotrophoblasts from term placenta [37], and choriodecidual explants [38]. The activation of MAP kinase signaling contributes to the activation of several transcriptional factors including NF-*κ*B, resulting in the upregulation of the genes of many proinflammatory proteins such as cytokines and chemokines [39]. The activation of NF-*κ*B can also increase the expression of COX and LOX, leading to the production of PGs and LTB_4_ [29]. Inflammatory cytokines, chemokines, PGs, and LTB_4_ have been reported to play essential roles in CAM-related preterm labor and birth [40]. Therefore, by inhibiting the activity of sEH—thus stabilizing endogenous EETs—AUDA likely exerts its anti-inflammatory effect via the attenuation of phosphorylation of MAP kinases and subsequent activation of NF-*κ*B in human gestational tissues with bacterial infection. Further studies are needed to verify our assumption and such results would be of clinical relevance, forming the basis of new therapeutic strategies in addition to antibiotic therapy in treating women with CAM.

## Figures and Tables

**Table 1:** Characteristics of the study population.

Variable	Normal pregnancy (*n* = 20)	Chorioamnionitis (*n* = 16)	*P*
Age (y)	35 (34-39)	36 (31-39)	0.56
Primiparity	9 (45%)	12 (75%)	0.07
Pregestational body mass index (kg/m^2^)	22.0 ± 3.0	23.0 ± 4.3	0.94
Gestational weight gain (kg)	11.0 ± 4.3	9.8 ± 5.9	0.20
Systolic blood pressure (mm hg)	117 ± 14	111 ± 16	0.11
Diastolic blood pressure (mm hg)	62 ± 10	65 ± 10	0.67
Gestational age (wk)	39.0 ± 1.0	37.0 ± 4.2	0.41
Birth weight (g)	3222 ± 453	3054 ± 990	0.51
Placental weight (g)	676 ± 148	627 ± 155	0.27
Maternal temperature (°C)	36 ± 0.4	38 ± 0.3	<0.001
Fetal baseline heart rate (bpm)	135 (130-145)	165 (160-175)	<0.001
White blood cell count (10^3^/*μ*L)	7.6 (6.7-9.2)	17.3 (14.7-19.0)	<0.001
Segmented neutrophil (%)	73 (69-78)	88 (84-90)	<0.001
Hemoglobin (g/dL)	12.0 ± 1.2	11.0 ± 1.8	0.31
Platelet count (10^3^/*μ*L)	221 (171-252)	222 (181-276)	0.56
C-reactive protein level (mg/dL)	—	13.0 (5.3-33.0)	NA
Microorganisms in amniotic fluid	—	7 (44%)	NA
1 − minute Apgar score < 7	0	1 (6%)	NA
5 − minute Apgar score < 7	0	1 (6%)	NA
Male fetus	12 (60%)	10 (63%)	0.88

Data presented as median (interquartile ranges), number (percentage), and mean ± standard deviation. *P* values based on the *χ*^2^ test, the Mann-Whitney *U*-test, or Student's *t*-test as appropriate. NA, not applicable for analysis.

## Data Availability

The data used to support the findings of this study are available from the corresponding author upon request.

## Supplementary Materials

Supplementary Materials Supplementary Figure 1: to determine the working concentration of LPS to examine its effect on the changes in sEH in human gestational tissues, we incubated villous explants with various concentrations of LPS (0, 1, 10, and 100 *μ*g/mL) for 24 h and measured the levels of IL-1*β* and IL-6 in the medium. As shown, LPS significantly increased the levels of IL-1*β* and IL-6 in the medium in a dose-dependent response; the difference was statistically significant even at the concentration of 1 *μ*g/mL and peaked at 10 *μ*g/mL. Therefore, LPS at a concentration of 10 *μ*g/mL was used to study its effect on the changes of transcription and translation of sEH in villous and fetal membrane explants in the subsequent experiments. Data are presented as mean ± SEM, *n* = 4 for each group. ^∗^*P* < 0.05 and ^∗∗∗^*P* < 0.001 compared with the vehicle controls; ^###^*P* < 0.001 compared with explants treated with LPS at a concentration of 1 *μ*g/mL, based on one-way ANOVA followed by Bonferroni's post hoc test. Supplementary Figure 2: after confirming the effect of LPS on the changes in sEH, we next studied the effect of AUDA on LPS-induced changes in levels of 14,15-DHET, IL-1*β*, and IL-6 in villous and fetal membrane explants. To determine the working concentration of AUDA, villous explants were incubated with LPS at a concentration of 10 *μ*g/mL and various concentrations of AUDA (0, 1, 10, and 100 *μ*M) dissolved in DMSO for 24 h and the levels of IL-1*β* and IL-6 in the medium were measured. As shown, the administration of AUDA at a concentration of 10 or 100 *μ*M significantly reduced the levels of IL-1*β* and IL-6 in the medium induced by LPS, with no difference in the effect between the two concentrations. Therefore, AUDA at a concentration of 10 *μ*M was used to study its effect on LPS-induced changes in 14,15-DHET, IL-1*β*, and IL-6 in villous and fetal membrane explants in the subsequent experiments. Data are presented as mean ± SEM, *n* = 4 for each group. ^∗∗∗^*P* < 0.001 compared with the vehicle control; ^###^*P* < 0.001 compared with explants treated with LPS (10 *μ*g/mL) alone, based on one-way ANOVA followed by Bonferroni's post hoc test. Supplementary Figure 3: representative blots of whole gel showing that the administration of LPS at a concentration of 10 *μ*g/mL to the culture medium led to a significant increase in the levels of sEH protein in villous explants (a), amnion (b), and choriodecidua (c) with the two-chamber culture system (*n* = 4 for each group). C57BL/6 mouse liver homogenate was used as the positive control for sEH (a). *β*-Actin was used to normalize loading variability. Molecular weight markers are shown at the left side of the blots.
