# Supplementary figures and images for: Increased Soluble Epoxide Hydrolase in Human Gestational Tissues from Pregnancies Complicated by Acute Chorioamnionitis
Source: Mediators Inflamm. 2019 Dec 5;2019:8687120. doi: 10.1155/2019/8687120 (PMC6915158; doi:10.1155/2019/8687120)

Supplementary Figure 1

**a**

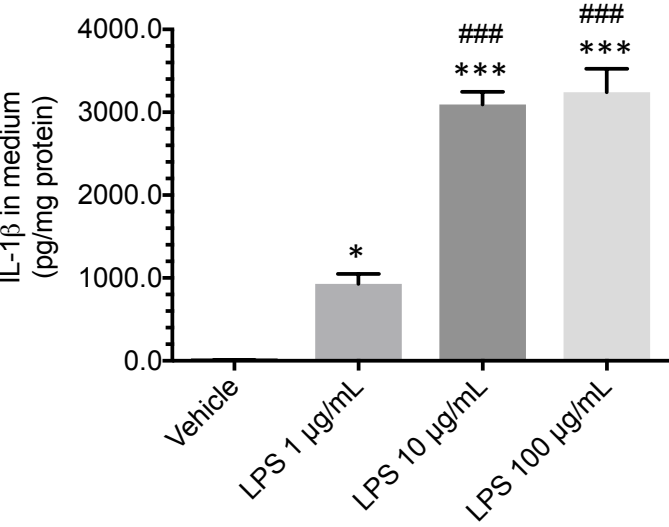

**b**

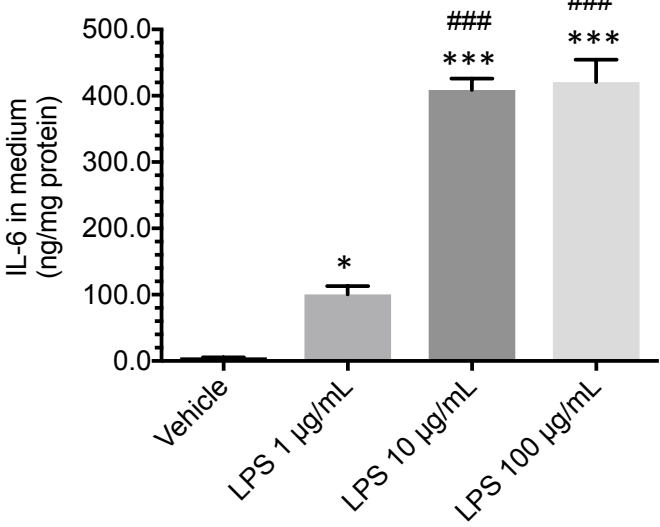

Supplementary Figure 2

**a**

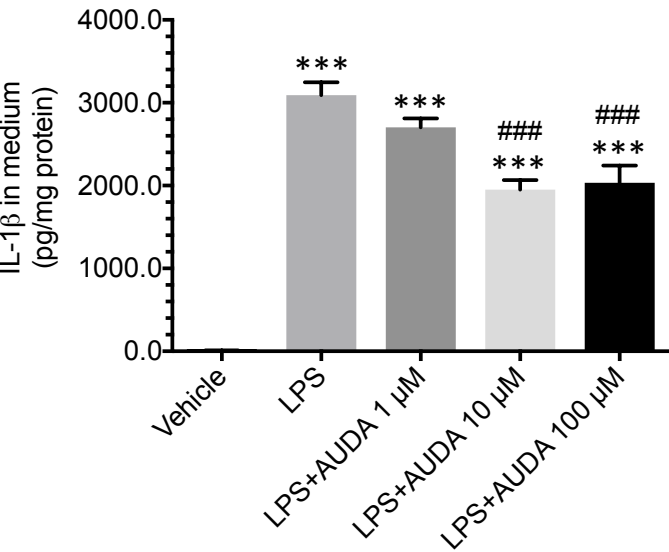

**b**

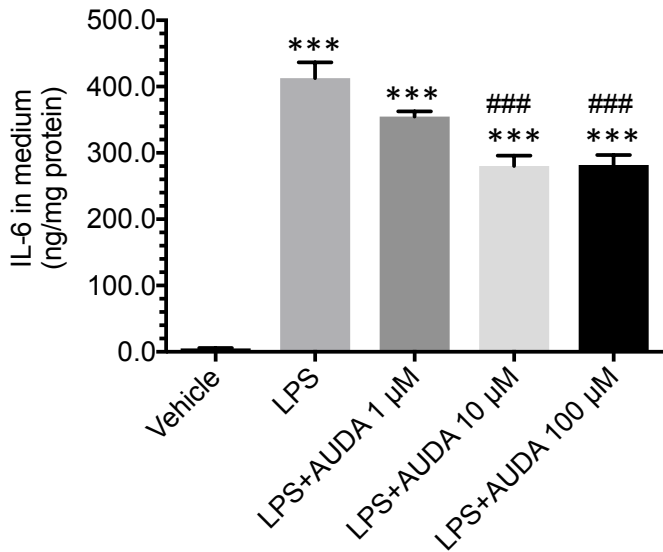

Supplementary Figure 3

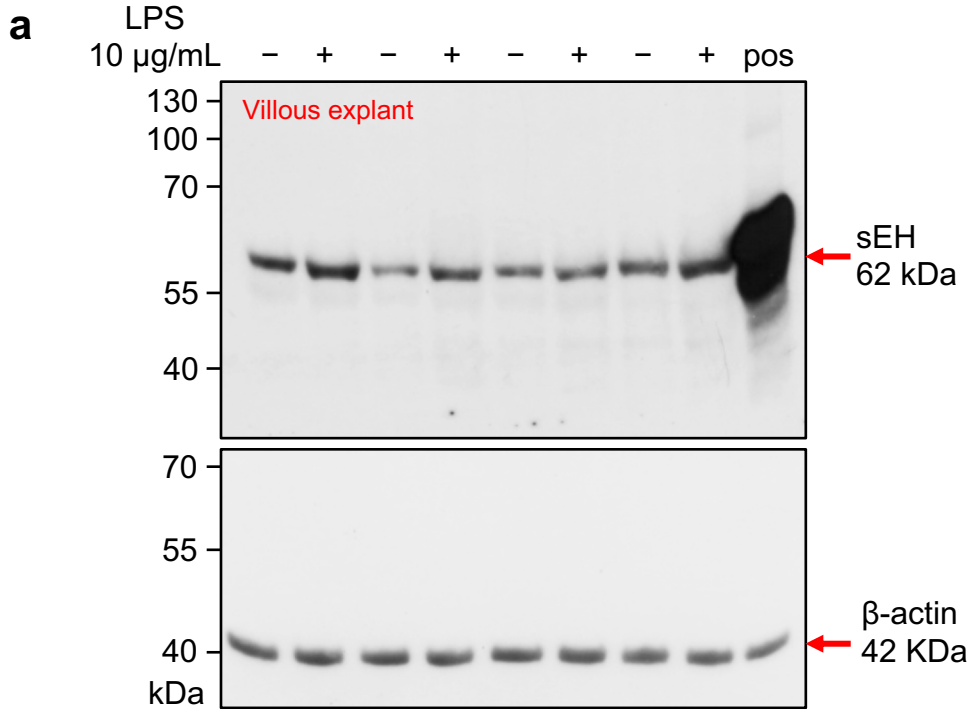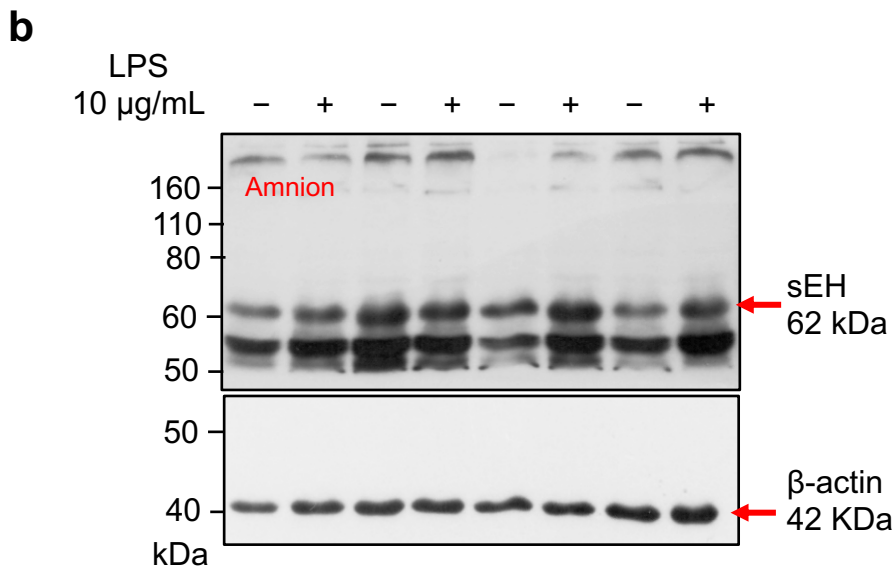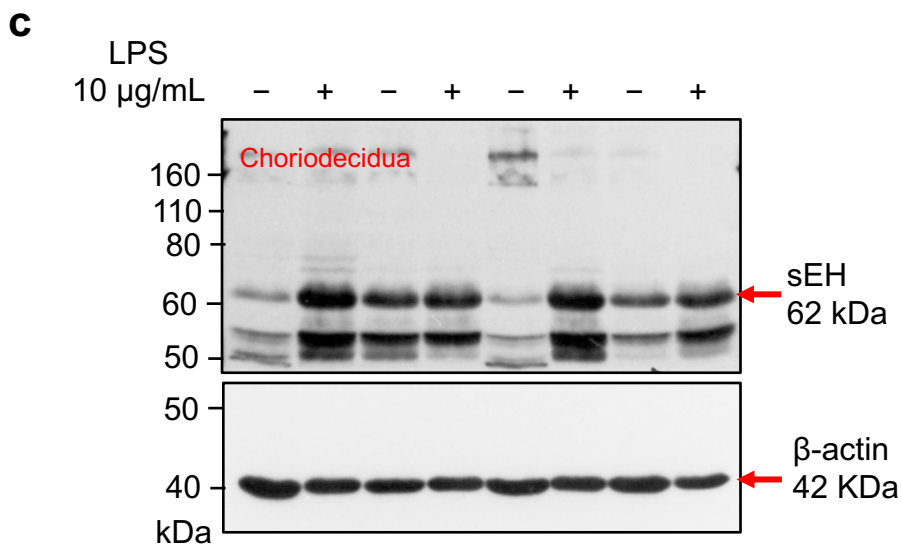

Supplement: Supplementary Materials — Supplementary Figure 1: to determine the working concentration of LPS to examine its effect on the changes in sEH in human gestational tissues, we incubated villous explants with various concentrations of LPS (0, 1, 10, and 100 μg/mL) for 24 h and measured the levels of IL-1β and IL-6 in the medium. As shown, LPS significantly increased the levels of IL-1β and IL-6 in the medium in a dose-dependent response; the difference was statistically significant even at the concentration of 1 μg/mL and peaked at 10 μg/mL. Therefore, LPS at a concentration of 10 μg/mL was used to study its effect on the changes of transcription and translation of sEH in villous and fetal membrane explants in the subsequent experiments. Data are presented as mean ± SEM, n = 4 for each group. ∗P < 0.05 and ∗∗∗P < 0.001 compared with the vehicle controls; ###P < 0.001 compared with explants treated with LPS at a concentration of 1 μg/mL, based on one-way ANOVA followed by Bonferroni's post hoc test. Supplementary Figure 2: after confirming the effect of LPS on the changes in sEH, we next studied the effect of AUDA on LPS-induced changes in levels of 14,15-DHET, IL-1β, and IL-6 in villous and fetal membrane explants. To determine the working concentration of AUDA, villous explants were incubated with LPS at a concentration of 10 μg/mL and various concentrations of AUDA (0, 1, 10, and 100 μM) dissolved in DMSO for 24 h and the levels of IL-1β and IL-6 in the medium were measured. As shown, the administration of AUDA at a concentration of 10 or 100 μM significantly reduced the levels of IL-1β and IL-6 in the medium induced by LPS, with no difference in the effect between the two concentrations. Therefore, AUDA at a concentration of 10 μM was used to study its effect on LPS-induced changes in 14,15-DHET, IL-1β, and IL-6 in villous and fetal membrane explants in the subsequent experiments. Data are presented as mean ± SEM, n = 4 for each group. ∗∗∗P < 0.001 compared with the vehicle control; ###P < 0. [file 8687120.f1.pdf]
